# Probiotics in piglet: from gut health to pathogen defense mechanisms

**DOI:** 10.3389/fimmu.2024.1468873

**Published:** 2024-11-04

**Authors:** Zipeng Jiang, Mingzhi Yang, Weifa Su, Liang Mei, Yuqi Li, Yuguang Guo, Yangyuan Li, Weifan Liang, Bo Yang, Zhiyi Huang, Yizhen Wang

**Affiliations:** ^1^ Guangdong VTR Bio-tech Co., Ltd, R&D Center, Zhuhai, China; ^2^ South China University of Technology, School of Biology and Biological Engineering, Guangzhou, China; ^3^ National Engineering Laboratory of Biological Feed Safety and Pollution Prevention and Control, Key Laboratory of Animal Nutrition and Feed, Ministry of Agriculture, Key Laboratory of Animal Nutrition and Feed Science of Zhejiang Province, Institute of Feed Science, Zhejiang University, Hangzhou, Zhejiang, China

**Keywords:** piglets, probiotics, pathogens, microbiota, intestinal barrier, mechanisms

## Abstract

Various problems and obstacles are encountered during pig farming, especially the weaning phase when switching from liquid to solid feed. Infection by pathogenic bacteria causes damage to the intestinal barrier function of piglets, disrupts the balance of the intestinal microbiota, and destroys the chemical, mechanical, and immune barriers of the intestinal tract, which is one of the main causes of gut inflammation or gut diseases in piglets. The traditional method is to add antibiotics to piglet diets to prevent bacterial infections. However, long-term overuse of antibiotics leads to bacterial resistance and residues in animal products, threatening human health and causing gut microbiota dysbiosis. In this context, finding alternatives to antibiotics to maintain pre- and post-weaning gut health in piglets and prevent pathogenic bacterial infections becomes a real emergency. The utilization of probiotics in piglet nutrition has emerged as a pivotal strategy to promote gut health and defend against pathogenic infections, offering a sustainable alternative to traditional antibiotic usage. This review introduces recent findings that underscore the multifaceted roles of probiotics in enhancing piglet welfare, from fortifying the gut barrier to mitigating the impacts of common bacterial pathogens. Meanwhile, this study introduces the functions of probiotics from different perspectives: positive effects of probiotics on piglet gut health, protecting piglets against pathogen infection, and the mechanisms of probiotics in preventing pathogenic bacteria.

## Introduction

The global livestock industry is undergoing a profound transformation, with the promotion of antibiotic-free husbandry emerging as a key trend in the development of the industry (1). The use of probiotics has been proven to significantly improve the gut health of piglets, both before and after weaning (2, 3). Weaning marks a significant turning point in the early life of piglets, signifying the transition from liquid to solid food and accompanying vulnerability to pathogenic microorganisms, which leads to the temporary disruption of gut barrier, digestive and absorptive disorder, and inflammation risks (4).

The gut microbiota plays a pivotal role in digestion, nutrient absorption, and immune function. Maintaining a balanced gut microbiota, intact gut barrier function, and a well-regulated immune system are hallmarks of a healthy gut in piglets. However, the immature microbiota of piglets cannot support them to defend the pathogens (5). Probiotics have emerged as a promising strategy to maintain gut health and combat bacterial infections, and the definition of probiotics had been revised in 2014 by the International Scientific Association for Probiotics and Prebiotics (ISAPP)—”live microorganisms that, when administered in adequate amounts, confer a health benefit on the host”. The roles of probiotics include enhancing nutrient absorption (6), reducing the incidence of diarrhea, promoting the development of a healthy gut microbiota (7), stimulating the production of beneficial metabolites (8), and supporting gut barrier and immune function integrity (9). In recent years, with the deepening understanding of the gut microbiome, probiotics have shown great potential in preventing and treating diseases in piglets caused by *Escherichia coli*, *Salmonella*, *Clostridium perfringens*, etc., which poses a serious threat to piglet health.

This review aims to provide a comprehensive overview of the probiotic application in the piglet stage, analyzing the mechanisms of action, functional characteristics, and positive effects of probiotics on the gut microbiota of piglets, and providing a scientific basis for the rational application of probiotics in pig farming, laying a theoretical foundation for promoting piglet health, improving farming efficiency, and ensuring food safety.

## The gut health of piglets

Gut health is a multifaceted concept that encompasses the maintenance of a balanced gut microbiota, an intact gut barrier, and a well-regulated immune system (10). A balanced gut microbiota is characterized by a diverse community of microorganisms that coexist in a stable state, contributing to the digestion of feed, the development of the immune system, and the overall health of the piglet (11).

## Microbiota balance

The pig’s gut hosts a diverse array of microorganisms, which include bacteria, fungi, and viruses. There are scholars who want to measure the gut microbiota of pigs on a temporal and spatial scale. For instance, analyzing the relative abundances of gut microbes in pigs, Hu et al. identified three core-predominant species: *Phascolarctobacterium succinatutens*, *Prevotella copri*, and *Oscillibacter valericigenes (* 12). Niu et al. identified a total of 22 phyla and 249 genera from fecal samples of pig, Firmicutes and Bacteroidetes were consistently the most dominant phyla, and as age increased, there was a notable rise in the proportion of *TM7* and *Tenericutes*, while *Lentisphaerae* and *Synergistetes* showed a decline (13). Another research reported that consistent with prior studies, Bacteroidetes and Firmicutes were the predominant bacterial phyla, with *Prevotella* and *Roseburia* being the most abundant genera (14). Among these studies, Firmicutes, Bacteroidetes, Actinobacteria, and Proteobacteria are the core phyla for pig microbiota.

In general, the compositional structure of intestinal microbiota corresponds to different functions, including maintaining nutritional, physiological, and immunological functions. Firstly, microbiota in pig gut help in the breakdown and absorption of nutrients, particularly complex carbohydrates that the host cannot digest alone. Microbiota across different segments of the intestine exhibit distinct metabolic capabilities for various nutrients. For instance, the microbiota in the proximal intestine primarily engages in amino acid metabolism, while that in the distal intestine is predominantly involved in fermenting dietary fiber (DF) (15). DF plays a crucial role in sustaining normal intestinal function. Its biological effects are primarily realized through microbial fermentation, predominantly in the distal small intestine and the large intestine, and short-chain fatty acids (SCFAs), the principal products of this fermentation process, serve as the primary energy source for intestinal cells (16). Secondly, the microbiota influences physiology by regulating metabolism, and this process is interactive (17). For example, microbial enzymes can modify bile acids, affecting lipid digestion and absorption, and certain bacteria produce amino acids and their derivatives, which can influence gut health and systemic processes or even the brain (18). Include compounds like bacteriocins, which have antimicrobial properties, and other bioactive molecules that may affect the host’s physiology (19). In conclusion, the gut microbiota of the pig is intimately linked to its functions, and its functioning is also related to its metabolites.

## Gut barrier function

The gut barrier function is a critical component of piglet gut health, which includes physical, chemical, biological, and immune barriers (20). The physical barrier is primarily composed of the intestinal epithelium and the tight junctions (TJs) between epithelial cells, which prevent the translocation of harmful substances and pathogens from the lumen into the bloodstream (21). Cells include the absorptive enterocytes (intestinal mucosal epithelial cell) and secretory enterocytes (enteroendocrine cell, goblet cell, and paneth cell), and intercellular junction complexes between intestinal epithelial cells, including TJs, adherens junctions, gap junctions, and bridges, are also important in maintaining the integrity of the epithelial barrier. The chemical barrier consists of secretions such as mucus, antimicrobial peptides (AMPs), and immunoglobulins that protect against pathogens. The mucus has many mucins including secretory mucin and binding mucin, covering the surface of the intestinal tract and defending against pathogens and other harmful substances, and AMPs in the chemical barrier are a class of peptides with antimicrobial activity, including defensins and lysozyme (10). Moreover, immunoglobulins such as secretory immunoglobulin A (sIgA) are essential for maintaining intestinal health; they not only defend against foreign pathogens, but also reduce pathogen attack on the intestinal epithelium by enhancing the intestinal barrier function (22). The biological barrier refers to the symbiotic relationship between the host and the commensal microbes that inhibit the growth of harmful bacteria (23). Studies have shown that the composition of intestinal microorganisms and the health of the body are closely related, such as some fiber-utilizing bacteria, which can break down indigestible fibers and insoluble proteins into monosaccharides and small peptides that can be absorbed by the animal to provide energy and nutrients for the organism, and a part of the bacteria that can produce SCFAs that can directly supply the body with energy and, at the same time, lower the pH in the intestine and inhibit the growth of pathogenic bacteria (24). With the intestinal epithelium, the mucus layer and innate immune cells constitute the immune barrier (25). A well-regulated immune system is capable of mounting an appropriate response to eliminate pathogens without causing excessive inflammation that could damage the gut tissue (26).

## Immune system

Specifically, the immune system plays a crucial role in gut health by recognizing and responding to both harmless commensal microbes and potential pathogens (27). Therefore, the function of the immune system is closely related to the gut microbes. Microbiota has the ability to regulate the immune function of host; it can stimulate the development and maturation of the immune system, including the gut-associated lymphoid tissue (GALT) (28). GALT is an important component of the mucosal immune system and plays a key role in regulating the immune response in the gut. It consists of components such as Peyer’s patches (PPs), mesenteric lymphoid nodes (MLNs), isolated lymphoid follicles, and lamina propria (29). As with long-term microbial colonization, sustained microbial exposure during the development of piglets is crucial for maintaining a balanced immune cell population (30). The functions of microorganisms in regulating the body’s immunity are also interconnected, for example, sIgA concentrations show a positive correlation with the abundance of Prevotella and are associated with enhanced animal growth (31). Meanwhile, SCFAs have immunomodulatory effects, which help prevent inflammation and microbial infections.

In piglets, the development of gut health is particularly important due to their underdeveloped immune systems and the significant changes in diet and environment they experience, especially during the weaning period. At this stage, the piglets are more susceptible to pathogens. Hence, the maintenance of gut health is a critical aspect of piglet rearing and overall health .

## Definition, classification, and function of probiotics

The definition of probiotics was introduced in 2014 and clearly defines the range of probiotics and non-probiotics (2), including three key attributes: live microorganisms, adequate quantities, and health benefits to the host. Recent developments have imposed stricter criteria on probiotic use, emphasizing safety and the absence of transferable antibiotic resistance genes or pathogenic virulence factors. Probiotics must also demonstrate resilience under normal gastrointestinal conditions, such as pH tolerance, exposure to proteolytic enzymes like trypsin, and bile salt tolerance.

Three types of probiotics are used as feed additives: Lactic acid bacteria (LAB) are characterized by their ability to ferment carbohydrates into lactic acid. According to Bergey’s Manual of Systematic Bacteriology, LAB is categorized into several genera including *Lactobacillus*, *Streptococcus*, *Bifidobacterium*, *Leuconostoc*, and *Pediococcus (* 32). These bacteria are typically acid-tolerant and bile-resistant and adhere strongly to surfaces (33). LAB prevent pathogen infection by lowering pH and competing for binding sites on the intestinal epithelium, while stimulating the immune system and preserving the gut barrier (34, 35). *Bacillus* species are Gram-positive, spore-forming rods capable of surviving under aerobic or anaerobic conditions. Their spores provide resistance to harsh environmental conditions. Known for heat tolerance, rapid germination, and enzyme secretion, *Bacillus* spores are used in processing, storage, and transportation, germinating into vegetative cells (36). Commonly used species in animal production include *Bacillus subtilis*, *Bacillus amyloliquefaciens*, *Bacillus natto*, *Bacillus licheniformis*, and *Bacillus coagulans (* 37). They maintain an anaerobic gut environment, inhibit pathogen proliferation, and produce organic acids and secrete enzymes. Additionally, they secrete secondary metabolites like bacteriocins and stimulate the host’s immune response (38).

**Figure 1:**
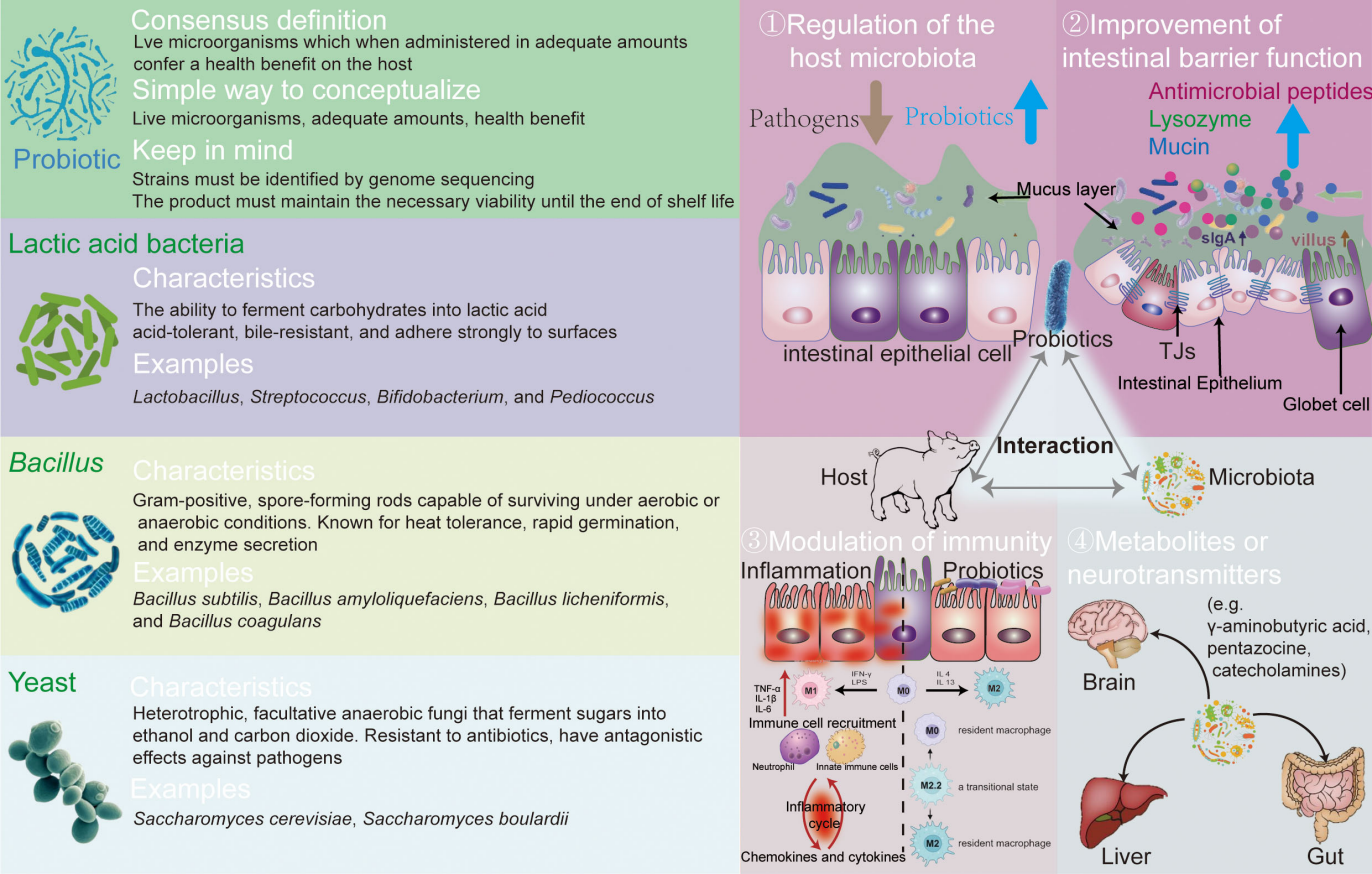
The definition, classification, and function of probiotics.

Yeast, primarily *Saccharomyces* species, are heterotrophic, facultative anaerobic fungi that ferment sugars into ethanol and carbon dioxide, used widely in brewing and food fermentation. As probiotics, yeasts offer nutritional benefits, rich in proteins and polysaccharides (39), and have antagonistic effects against pathogens, improving gut ecology (40). Yeast cell wall extracts, containing mannans and glucans, boost gut immunity (41). However, the efficacy of live yeast and yeast culture varies significantly in animal production, influenced by factors such as species and rearing conditions. The left part of **Figure 1** overviewed the probiotics knowledge, introducing the main characteristics of three types of probiotics.

## Effects of probiotics on piglet gut health

The strategic use of probiotics can effectively address common challenges faced during the piglet stage, such as stress, diarrhea, disrupted gut microbiota, and reduced growth rates. **Table 1** summarized the impact of probiotics on the intestinal health of piglets. Liu et al. demonstrate the effectiveness of a combination of *Lactobacillus casei* and *Enterococcus faecalis* (in a 3:1 ratio, 1×10^9^ CFU/mL) administered orally to nursing piglets on day 1 (1 mL), day 7 (2 mL), day 14 (3 mL), and day 21 (4 mL). This probiotic mix was found to decrease the numbers of *E. coli* and *C. perfringens* in the intestines compared to the control group (42). Similarly, using *B. amyloliquefaciens* 1 mL (1×10^8^ CFU/mL) continuously for 15 days resulted in a significant reduction of *Escherichia*, *Shigella*, and *Streptococcus*, while increasing the abundance of SCFA-producing bacteria and enhancing the antioxidant capacity of the piglets (43). Varun et al. administered *Pediococcus* FT28 1 mL (1×10^9^ CFU/mL) to piglets from day 7 to day 21, observing an improvement in intestinal integrity and a significant reduction in mortality rates (44). Richard et al. indicated that early supplementation of probiotics (*Bifidobacterium animalis*, *Lactobacillus acidophilus*, and *L. casei*, 5×10^9^ CFU/3 mL), can alleviate susceptibility to necrotizing enterocolitis (NEC) in piglets, as well as reduce *C. perfringens* (45). *E. faecalis* (2.5×10^6^ CFU/g diet) was used to treat the weaned piglet for 28 days, which decreased diarrhea rate and improved the growth performance (46). 

**Table 1:** Summary of effects of probiotics on piglet gut health.

Microorganism category	Microorganism name	Treatment	Host health influence	Reference
Lactic acid bacteria	*Lactobacillus casei, Enterococcus faecalis*	Oral gavage (*Lactobacillus casei*: *Enterococcus faecalis* = 3:1, 1×10^9^ CFU/mL) to piglets on day 1 (1 mL), day 7 (2 mL), day 14 (3 mL), and day 21 (4 mL)	Reduction of intestinal pathogenic *Escherichia coli* and *Clostridium perfringens* in piglets	(42)
	*Pediococcus*	Piglets were gavaged continuously with probiotics 1 mL (1×10^9^ CFU/mL) from 7 days after birth to day 21	Significantly improves intestinal integrity and significantly reduces mortality in lactating piglets	(44)
	*Bifidobacterium animalis, Lactobacillus acidophilus, Lactobacillus casei*	28 prematurely born piglets were fed 5 × 10^9^ CFU/3 mL of breast milk supplemented with the probiotic combination until weaning	May alleviate intestinal dysfunction and NEC susceptibility in piglets and reduce *Clostridium perfringens* abundance	(45)
	*Lactobacillus reuteri, Lactobacillus fermentum, Lactobacillus casei*	Feeding piglets at 7.74 ± 0.4 to 8.4 ± 0.5 log (CFU/g) until weaning	Detection of fecal load of pathogenic microorganisms at weaning showed that probiotic addition significantly reduced *Escherichia coli* abundance and *Clostridium perfringens* abundance at weaning; addition of reuterin alone was less effective	(51)
	*Enterococcus faecalis*	Piglets weaned at 26 days of age were fed basal diet supplemented with *Enterococcus faecalis* (2.5 ×10^6^ CFU/g of feed) for 28 days	Decreasing diarrhea index and improved growth performance, increasing the relative number of *Lactobacillus* in feces	(46)
*Bacillus*	*Bacillus amyloliquefaciens*	Gavage of piglets for 15 consecutive days from birth (1 × 10^8^ CFU/mL/day)	Significantly reduces the abundance of pathogenic bacteria (*Escherichia coli*, *Shigella*, *Streptococcus*, etc.) in piglets, while increasing the abundance of SCFA-producing bacteria, and significantly improves the antioxidant capacity of piglets	(43)
	*Bacillus licheniformis*	Piglets were fed *Bacillus licheniformis* (5×10^5^–1×10^6^ CFU/g) for 28 consecutive days from birth	Significantly reduces the rate of diarrhea in weaned piglets, improves their antioxidant and immune functions, while maintaining their intestinal flora structure	(48)
	*Bacillus amyloliquefaciens*	Feed containing 2×10^5^ CFU/g *Bacillus amyloliquefaciens* was fed to piglets for 28 consecutive days	The *Bacillus amyloliquefaciens* group significantly improved the intestinal barrier and immune function in piglets compared to the antibiotic group	(49)
	*Bacillus coagulans*	Feed containing *Bacillus coagulans* of feed (4.8×10^6^ CFU/g) was fed to piglets for 26 days	Compared with the antibiotic group, it can significantly reduce the rate of diarrhea in piglets and, at the same time, improve piglet production performance and the structure of the intestinal flora of piglets	(50)
	*Bacillus-Complex Probiotics*	For piglets 0–21 days, add probiotic complex (>1.0 × 10^12^ CFU/g) to feed at 0.1% (1×10^9^ CFU/g), 0.2% (2×10^9^ CFU/g), and 0.3% (3×10^9^ CFU/g)	Reduces *Escherichia coli* in piglets and reduces ammonia emissions while improving pig nutritional digestibility	(101)
	*Bacillus subtilis*	Weaning pigs basal diet supplemented with 4×10^6^ CFU/g feed of *B. subtilis* KN-42	Increasing average daily gain (ADG) and feed efficiency of piglets and decreasing diarrhea index and the relative number of *Escherichia coli*	(102)
Yeast	*Rhodotorula mucilaginosa*	Oral administration of 1×10^10^ CFU/mL/day to piglets for four consecutive weeks from birth	Significantly improves the growth performance and promotes the development of their intestinal tract to resist the invasion of harmful bacteria, as well as improving their antioxidant capacity	(47)
	*Saccharomyces cerevisiae*	Piglets are fed yeast (0.05% in phase 1, 0.025% in phase 2, and 0% in phase 3) continuously for 24 days from birth to weaning	Decreased antibiotic resistance of *Escherichia coli* in piglet feces while increasing piglet performance	(52)
	*Saccharomyces cerevisiae*	Weaning pigs basal diet supplemented with live yeast (12.9×10^7^ CFU/g of feed) *Saccharomyces cerevisiae* for 21 days	Decreasing *Escherichia coli* in the ileum and cecum contents; increased serum SOD activity and jejunum mucosal SIgA secretions	(53)
Three types of mixed bacteria	*Enterococcus faecium, Bacillus subtilis, Saccharomyces cerevisiae*, and *Lactobacillus paracasei*	Piglets weaned at 28 days of age were fed the basal diet mixed with the probiotics (>1×10^8^ CFU/g for each strain) for 21 days	Increasing fecal acetic acid and propionic acid; increasing growth performance and significantly reducing the weaning stress	(54)

Probiotics also offer a promising solution to mitigate the challenges following weaning, such as intestinal dysfunction and diarrhea. Hu et al. administered *Rhodotorula mucilaginosa* at a concentration of 1×10^10^ CFU/mL/day for 4 weeks to weaned piglets, and results were shown to significantly improve the piglets’ performance, promote gut development, protect against harmful bacteria, and enhance antioxidant capacity (47). Yu et al. fed piglets a diet supplemented with *B. licheniformis* (5×10^5^–1 × 10^6^ CFU/g of feed) for 28 days. The results indicated that *B. licheniformis* significantly reduced diarrhea in weaned piglets, improved their antioxidant and immune functions, and maintained the structure of the gut microbiota (48). Du et al. provided piglets with a diet containing 2×10^5^ CFU/g of *B. amyloliquefaciens* SC06 for 28 days. Compared to the antibiotic group, the SC06 group exhibited a significant enhancement in the piglets’ gut barrier and immune functions (49). Sun et al. fed piglets with a diet containing 600 g/ton of *B. coagulans* for 26 days. The results showed that, compared to the antibiotic group, *B. coagulans* (4.8×10^6^ CFU/g of feed) significantly reduced the diarrhea rate in piglets, improved their performance, and optimized the gut microbiota structure (50). Wang et al. added a mixture of probiotics (*Lactobacillus reuteri*, *Lactobacillus fermentum*, and *L. casei*) and probiotics-derived antimicrobial compounds (reuterin) to the piglet feed at concentrations ranging from 7.74 ± 0.4 to 8.4 ± 0.5 log (CFU/g). The addition of probiotics significantly reduced the abundance of *E. coli* and *C. perfringens* at weaning, whereas the reuterin had less pronounced effects (51). Jenna et al. added yeast-based probiotics yeast (0.05% in phase 1, 0.025% in phase 2, and 0% in phase 3) to the piglet diet and observed, after continuous feeding for 24 days, a decrease in the antibiotic resistance of *E. coli* in the feces and an increase in the growth performance (52). Zhu et al. fed weaning pigs with *Saccharomyces cerevisiae* (12.9×10^7^ CFU/g of feed) for 21 days, and they found that the *E. coli* decreased, and SOD activity and sIgA secretions increased (53). One research utilized the three types of mixed bacteria including LAB*, Bacillus*, and yeast (>1×10^8^ CFU/g for each strain), which increased the growth performance and fecal acetic and propionic acid of weaned piglets, and reduced the diarrhea (54).

Moreover, probiotics can also have an impact on GALT, which has important implications for the host immune system and gut health. In weaned piglets, DNA from *B. animalis* was detected in MLN and PPs, with concentrations increasing proportionally to the administered probiotic dose (55). Consumption of *Lactobacillus plantarum* Lp6 was found to modulate gene expression within the jejunal PPs, and this modulation affected genes associated with immune response, cell differentiation, etc (56). One research used fermented milk with *Lactobacillus bulgaricus* and *Streptococcus thermophilus* to test its function, with results indicating that probiotics modulated the gut microbiota but did not clearly exert beneficial effects on GALT lymphocyte cell numbers and mucosal IgA levels (57). In addition to these direct effects, there is some evidence that probiotics stimulate the expression of inflammatory factors through GALT (58). For instance, O’Mahony et al. reported that commensal bacteria, such as *Lactobacillus* and *Bifidobacterium*, induced the production of regulatory cytokines, including IL-10, by MLN and MLN-derived dendritic cells (DCs); in contrast, pathogenic bacteria like *Salmonella* induced the production of Th1-polarizing cytokines (IL-12 and TNF-α) (59). *Bifidobacteria* from VSL#3 have the most pronounced anti-inflammatory effects during the maturation process of DCs, and they up-regulate the production of IL-10 by DCs, reduce the expression of costimulatory molecules such as CD80 and CD40, and decrease the production of IFN-γ by T cells (60). Using *Lactobacillus jensenii* also modulated the GALT-associated part, increasing the expression of T cell-related mRNA (CD3, IL-2, and IFN-γ) (61). Furthermore, some probiotics and commensal bacteria can activate local antigen-presenting cells, thereby enhancing antigen presentation to B lymphocytes and boosting the production of sIgA both locally and systemically (62). The whole activation of GALT strengthens the host’s defenses against pathogens and improve the gut health.

In this study, we collectively highlight the potential benefits of probiotics in the gut health and growth performance with piglets, demonstrating their roles in reducing diarrhea, enhancing immune and antioxidant functions, and optimizing gut microbiota composition. The right part of **Figure 1** demonstrates the main functions of probiotics: (1) regulation of the host microbiota, improving the beneficial bacteria; (2) improvement of intestinal barrier function and competitive exclusion of pathogens; (3) modulation of immunity, accelerating the maturation of gut microbiota; and (4) secretion of metabolites or neurotransmitters, producing antimicrobial substances.

## Probiotics and prevention of pathogenic bacterial infections

Probiotics have been shown to regulate gut health, but are they also effective during pathogen infections, especially during the formative “window period” of gut microbiota establishment in piglets? This query now stands as a focal point for scholarly investigation.

**Table 2** summarizes the impact of probiotics on preventing pathogens. Qin et al. utilized *L. casei* to prevent enterotoxigenic *E. coli* (ETEC) K88 infection in piglets. They revealed that probiotics effectively alleviated symptoms associated with ETEC infection, such as intestinal damage and increased diarrhea rates, and activated mucosal and humoral immune responses in the piglets (63). Prasert et al. fed piglets a diet supplemented with 3 mL of *L. plantarum* and *Lactococcus lactis* (1×10^9^ CFU/mL). After ETEC challenge (5×10^9^ CFU/mL) 14 days post-weaning, they observed that probiotics improved gut microbiota composition, reduced antibiotic resistance in the gut microbiota, and mitigated oxidative stress (64). Guevara Ordaz et al. continuously fed piglets with *L. plantarum* (2*×*10^10^ CFU/animal/day) from 4 to 6 weeks of age. Following single 6-mL oral dose ETEC K88 (2×10^9^ CFU/mL) challenge from days 23 to 27, they noted an increased abundance of *L. plantarum*, along with a reduction in diarrhea (65). Yang et al. administered a mixture of 10 mL of *B. subtilis* and *B. licheniformis* (3.9×10^7^–7.8×10^7^ CFU/mL) to piglets for 36 days. Post-challenge with 10 mL ETEC F4 (1.0×10^9^ CFU/mL) on day 21, they observed a significant decrease of *E. coli (* 66). Zhang et al. investigated the preventive effects of *Lactobacillus rhamnosus* GG 10 mL dose (1×10^10^ CFU/mL) against ETEC K88 10 mL dose (1×10^10^ CFU/mL) infection in weaned piglets. They found that the incidence of diarrhea was significantly lower in the probiotic group, and enhanced secretion of sIgA (67). Bhandari et al. evaluated the efficacy of *B. subtilis* (1.2×10^6^ CFU/g of feed) as a direct-fed microbial in preventing ETEC K88 infection in weaned piglets (challenged at day 24, with 6 mL, 6.4×10^9^ CFU/mL). Their results indicated that probiotics were equally effective as antibiotics in preventing diarrhea in piglets (68). Ahmed et al. conducted a study using *Lactobacillus* and *Bacillus* as probiotics (3.2×10^6^ CFU/g of feed) to prevent 5-mL *Salmonella* KCTC 2515 (5.9×10^8^ CFU/mL) and *E. coli* KCTC 2571 (2.3×10^8^ CFU/mL) infections in piglets, and results showed that probiotic prevention led to a significant reduction in *E. coli* and *Streptococcus (* 69). Emili Barba et al. utilized a *Bifidobacterium longum* subsp. *infantis* CECT 7210 2-mL dose (1×10^9^ CFU) to prevent *Salmonella typhimurium* 2-mL dose (5×10^8^ CFU) infection in weaned piglets. They found that probiotics resulted in reduced shedding of *Salmonella*, lower diarrhea scores, and enhanced gut immunity (70). Casey et al. administered a probiotic mixture (4×10^9^ CFU) to weaned piglets for 30 consecutive days, followed by a *Salmonella typhimurium* (1×10^8^ CFU) challenge on the sixth day. The study revealed that animals treated with probiotics had significantly reduced incidence, severity, and duration of diarrhea (71). Upadhaya et al. found, in a 3-week probiotic intervention using *Bacillus* (1×10 ^9^ CFU/g) in weaned piglets challenged with *Salmonella typhimurium* 1 mL (1×10^11^ CFU/mL), that probiotic increased *Lactobacillus* and exerted immunomodulatory effects (72). Trevisi et al. fed piglets *S. cerevisiae* (5×10^7^ CFU/g of feed) at weeks 4 and 7. After the ETEC F4 1.5 mL (1×10^8^ CFU/mL) challenge on day 24, they found a substantial reduction of ETEC (73). *S. cerevisiae boulardii* was used to prevent the lipopolysaccharide (LPS, 25 μg/kg BW) challenge in weaning pigs, and results showed that the growth performance increased and the LPS-induced mortality was reduced obviously (74). Weaning piglets were fed with *S. cerevisiae* to defend the ETEC K88 6 mL (5×10^10^ CFU/mL), and a study illustrated that it significantly reduces the diarrhea scores and increases the IgA levels (75).

**Table 2:** Summary of effects of probiotics on protecting against pathogens.

Microorganism category	Microorganism name	Treatment	Host health influence	Reference
Lactic acid bacteria	*Lactobacillus casei*	Prevention of *enterotoxin-producing Escherichia coli* (ETEC) K88 infection in piglets, and pLA-ETEC K88/*L. casei* (5 × 10^11^ CFU/mL) were orally administered daily on days 0–5	Probiotics can effectively alleviate the symptoms of intestinal damage and increased diarrhea caused by ETEC, and *Lactobacillus casei* can effectively activate the mucosal and humoral immune response in piglets	(63)
	*Lactobacillus plantarum and Lactococcus lactis*	Feeding probiotics 3 mL LAB (1×10^9^ CFU/mL) until 14 days after weaning for ETEC infection (5×10^9^ CFU/mL) in piglets	Probiotic treatment improves gut microbial communities and reduces antibiotic resistance in gut microbes, as well as mitigating ETEC-induced gut oxidative stress in piglets	(64)
	*Lactobacillus plantarum*	Piglets were fed continuously with *Lactobacillus plantarum* (2*×*10^10^ CFU/animal/day) for 4 weeks to 6 weeks and attacked with a single 6-mL oral dose of ETEC K88 (2×10^9^ CFU/mL) on days 23 to 27	Increased abundance of *Lactobacillus plantarum* in the ileum and colon of piglets, together with attenuation of diarrhea due to ETEC	(65)
	*Lactobacillus rhamnosus LGG*	Feeding probiotics 10 mL dose (1×10^10^ CFU/mL) until piglets are weaned, followed by an offensive treatment with *Escherichia coli* K88 10 mL dose (1×10^10^ CFU/mL)	The incidence of diarrhea was significantly reduced in the probiotic group, while regulating the intestinal microbiota of piglets and increasing the secretion of immunoglobulin sIgA in the jejunum and ileum	(67)
	*Lactobacillus* and *Bacillus*	Prevention of 5 mL of *Salmonella KCTC* 2515 (5.9×10^8^ CFU/mL) and *Escherichia coli* KCTC 2571 (2.3×10^8^ CFU/mL) infections in piglets after probiotic treatment (3.2×10^6^ CFU/g diet)	Prevention with probiotics resulted in significant reductions in *Escherichia coli* and *Streptococcus*, which significantly improved piglet physical status compared to the infected group	(69)
	*Bifidobacterium infantis* subspecies *CECT 7210*	Using probiotics 2 mL dose (1×10^9^ CFU) and *Salmonella typhimurium* 2 mL dose (5×10^8^ CFU) infection in weaned piglets	Probiotics treatment resulted in reduced fecal excretion of *Salmonella*, lower diarrhea scores, and increased intestinal immunity in piglets	(70)
	*Lactobacillus, Lactobacillus salivarius, Pediococcus pentosaceus*	Weaned piglets were continuously gavaged probiotics for 30 days (4×10^9^ CFU) and then attacked with *Salmonella typhimurium* (1×10^8^ CFU) on the sixth day	Significant reduction in diarrhea incidence, severity, and duration of bacteria in probiotic-treated animals	(71)
	*Lactobacillus plantarum*	Young piglets challenged with ETEC K88 (1×10^8^ CFU per pig) and piglets were fed with *Lactobacillus plantarum* (5×10^7^ CFU/g diet)	Improving performance and effectively preventing diarrhea; enhancing the function of the intestinal barrier through the protection of intestinal morphology, maintenance of intestinal permeability, and regulation of tight junction (TJ) protein gene expression	(103)
*Bacillus*	*Bacillus subtilis* and *Bacillus licheniformis*	Piglets were fed probiotics 10 mL (3.9×10^7^–7.8×10^7^ CFU/mL) for 36 days and were attacked with ETEC F4 10 mL (1.0×10^9^ CFU/mL) on the 21st day	Probiotic treatment can significantly reduce the abundance of *Escherichia coli* in feces	(66)
	*Bacillus subtilis*	Preventing the effect of *Escherichia coli* K88 (attacking 6 mL 6.4 × 10^9^ CFU/mL at 24 days of age) in weaned piglets by *Bacillus subtilis* (1.2×10^6^ CFU/g of feed)	Probiotics and antibiotics are equally effective in preventing diarrhea in piglets	(68)
	*Bacillus subtilis*	A 3-week *Bacillus* (1×10^9^ CFU/g) treatment to investigate the role of prevention of *Salmonella typhimurium* 1 mL (1×10^11^ CFU/mL) in weaning piglets	Probiotic supplementation increased Lactobacillus counts in piglets’ feces and had some positive immunomodulatory effects	(72)
Yeast	*Saccharomyces cerevisiae*	Piglets were fed probiotics (5×10^7^ CFU/g of feed) at weeks 4 and 7 and attacked on day 24 with *Escherichia coli* ETEC F4 1.5 mL (1×10^8^ CFU/mL)	The results of the experiment can be found to significantly reduce the ETEC level	(73)
	*Saccharomyces cerevisiae*	Weaning pigs orally challenged with ETEC K88 (1.5×10^11^ CFU/piglet) and pigs were fed with live yeast *Saccharomyces cerevisia*e (1×10^7^ CFU/g diet)	Significantly reducing the daily diarrhea scores, and shedding of pathogenic ETEC bacteria in feces and increasing IgA levels in the serum of piglets	(104)
	*Saccharomyces cerevisiae*	*Saccharomyces cerevisiae* fermentation products were used to prevent ETEC K88 6 mL (5×10^10^ CFU/mL) infection in weaning pigs	Low abundance of ileal mucosa-adherent ETEC K88 and prevalence of *Enterobacteriaceae* in ileal digesta	(75)
	*Saccharomyces cerevisiae boulardii*	Weaning pigs orally challenged with *Escherichia coli* lipopolysaccharide (LPS, 25 μg/kg BW) were treated with *Saccharomyces cerevisiae boulardii*	ADG increased significantly by 39.9% and LPS-induced piglet mortality was reduced obviously	(74)
	*Saccharomyces cerevisiae*	Weaning pigs challenged with *Salmonella* (1×10^9^ CFU) were fed *Saccharomyces cerevisiae* fermentation products	Increasing compensatory body weight gains after *Salmonella* infection and increasing *Salmonella* shedding in feces	(105)

In summary, the application of probiotics can alleviate disturbances caused by bacterial diseases such as those from *E. coli* and *Salmonella*. The studies mentioned here illustrate the ability of probiotics to mitigate the impacts of bacterial pathogens on piglets, emphasizing their roles in enhancing gut barrier integrity, regulating the gut microbiota, and modulating immune responses. How exactly do probiotics prevent pathogenic bacterial infections (which is also currently the dominant line of research)?

## Molecular mechanisms of probiotics against pathogenic bacteria

On a macro level, probiotics can counteract the negative consequences caused by pathogenic bacteria. At the molecular level, studies demonstrate that these beneficial microbes wield their effects through various mechanisms. For instance, they can modulate the host’s immune system, and probiotics also directly influence other microorganisms in the gut. Additionally, they are able to secrete small molecules or metabolites that inhibit the proliferation and virulence of pathogens.

Probiotics exert regulatory influence on both the innate and adaptive immunity of the host, affecting diverse cell types including macrophages, DCs, and T and B lymphocytes. They stimulate immune responses, enhancing overall host immunity and protecting against intestinal diseases (27). Probiotics prompt DCs to secrete anti-inflammatory cytokines, such as IL-10, triggering anti-inflammatory responses (76). The reduction in pro-inflammatory cytokine release by immune cells is attributed to probiotics’ interference with inflammatory signaling pathways, notably NF-κB, MAPK, and MLCK (77). Activation of these pathways leads to excessive release of pro-inflammatory cytokines, compromising the integrity of the gut barrier. Pathogenic bacteria activate the NF-κB and MAPK pathways, promoting the release of cytokines like IL-1β, IL-6, and IL -8, which recruit neutrophils to infected sites, causing inflammatory tissue damage (78). Xie et al. demonstrated that *L. reuteri* CO21 increases the anti-inflammatory capacity in piglets by inhibiting NF-κB and MLCK pathways, mitigating inflammation induced by ETEC K88 (79). Zhou et al. found that *L. plantarum* attenuates the inflammation mediated by *C. perfringens* through the modulation of MAPK and NF-κB pathways (80). *B. subtilis* BS29784 was shown to prevent the nuclear translocation of NF-κB by blocking IκB degradation, thereby limiting the accumulation of pro-inflammatory cytokines like IL-1β (38). *L. reuteri* was found to alleviate ETEC K88-induced inflammation by inhibiting the activation of the MLCK pathway, thus reducing inflammation and enhancing epithelial function (81). Probiotics also play a role in stimulating the production of gut antibodies, impacting the gut barrier by promoting IgA from B cells (82). Gut IgA interferes with pathogen adherence to the epithelium (83), and studies have shown that *Saccharomyces boulardii* and *L. rhamnosus* enhance sIgA levels or immunoglobulin secretion (84, 85). Moreover, probiotics can modulate the host’s immune response by influencing macrophage phagocytosis of pathogens. For example, *L. casei* exhibits enhanced inhibition of pathogenic *Pseudomonas aeruginosa* and *Listeria monocytogenes* in the gut, correlated with increased macrophage activity (86).

The direct impact of probiotics on commensal and pathogenic bacteria is another mechanism by which they resist pathogen infection, as probiotics can directly influence the gut microbiota. Gordon et al. showed that introducing probiotics into the mouse gut alters the metabolic pathways of its indigenous microbiome (87). Certain bacteria may find their ability to compete for nutritive or non-nutritive substance, such as iron, altered by the presence of probiotic strains, preventing pathogenic microorganisms from proliferating. For instance, *L. acidophilus* can utilize iron in the gut, thereby limiting its availability for other pathogens (88). Gordon et al. also demonstrated that treating germ-free mice with *Bifidobacteria* and *Lactobacillus* resulted in shifts in gene expression patterns, showing enhanced carbohydrate utilization capabilities by the bacteria. Interestingly, the sets of genes that responded to each probiotic did not overlap, indicating distinct effects on the microbiome (87). Probiotics can also antagonize pathogens directly, co-aggregating with them to prevent adhesion and colonization. Studies have shown that LAB can enhance their own colonization ability by self-aggregation and co-aggregation, forming a barrier against pathogen colonization (89, 90). Research has found that *Bifidobacterium lactis* or *L. rhamnosus* GG can reduce the attachment of pathogens like *Salmonella*, *C. perfringens*, and *E. coli* to porcine intestinal mucus (9). This anti-adhesive effect is achieved by competing for the same receptors as the pathogens.

Probiotics combat pathogenic bacteria through the secretion of various small-molecule substances that exhibit antimicrobial properties. These include bacteriocins, SCFAs, organic acid, and hydrogen peroxide. Bacteriocins, ribosomally synthesized AMPs, bind to microbial cells and disrupt phospholipid membranes, causing cellular leakage and inhibiting DNA/RNA synthesis and cell wall protein production (91). The probiotic *B. subtilis* PB6 produces surfactin, which restrains *C. perfringens* colonization. *B. amyloliquefaciens* RX7 has been shown to produce antibiotics with broad-spectrum antibacterial and antifungal activities, effectively curbing the growth of *Candida albicans (* 92). Bacteriocins produced by *Bifidobacterium* NCFB exhibit activity against a range of Gram-positive bacteria, owing to their ability to adhere to bacterial surfaces (93, 94). Piewngam et al. observed that *Bacillus* could successfully defend against *Staphylococcus aureus* infection, primarily by reducing pathogen numbers through bacteriocin production (95). Secondary metabolites from bacteria can also inhibit quorum sensing (QS) in pathogens, disrupting their communication and virulence generation. For instance, pathogens equipped with QS systems, such as *S. aureus* and *C. perfringens*, communicate via the Agr system. *Bacillus* species capable of producing lipopeptides like fengycins (e.g., *B. subtilis* and *B. amyloliquefaciens*) can interfere with Agr signaling in *S. aureus*, disrupting QS system and preventing colonization in the gut or nasal cavity (96). Similar findings were reported by Piewngam et al. in a murine study, where oral administration of *B. subtilis* spores inhibited *E. faecalis* translocation from the gut to the bloodstream and systemic infection, with the mechanism involving fengycins and surfactant protein lipopeptides (97). Organic acids, particularly acetic and lactic acid, produced by probiotics can inhibit the growth of many enteric pathogens. Undissociated lactic acid acts as a permeabilizer for Gram-negative bacterial membranes, leading to cell disruption upon entry into the cytoplasm. It can also lower the intracellular pH, potentiating the effects of other antimicrobial compounds (98, 99). Hydrogen peroxide (H_2_O_2_) is another antimicrobial mechanism utilized by commensal bacteria. H_2_O_2_ can attenuate pathogen virulence, reduce invasion of epithelial cells, and alter gene transcription and signal transduction (100). Probiotic strains with H_2_O_2_-producing traits have been shown to significantly inhibit *Salmonella typhimurium* and *S. aureus in vitro*.

**Figure 2:**
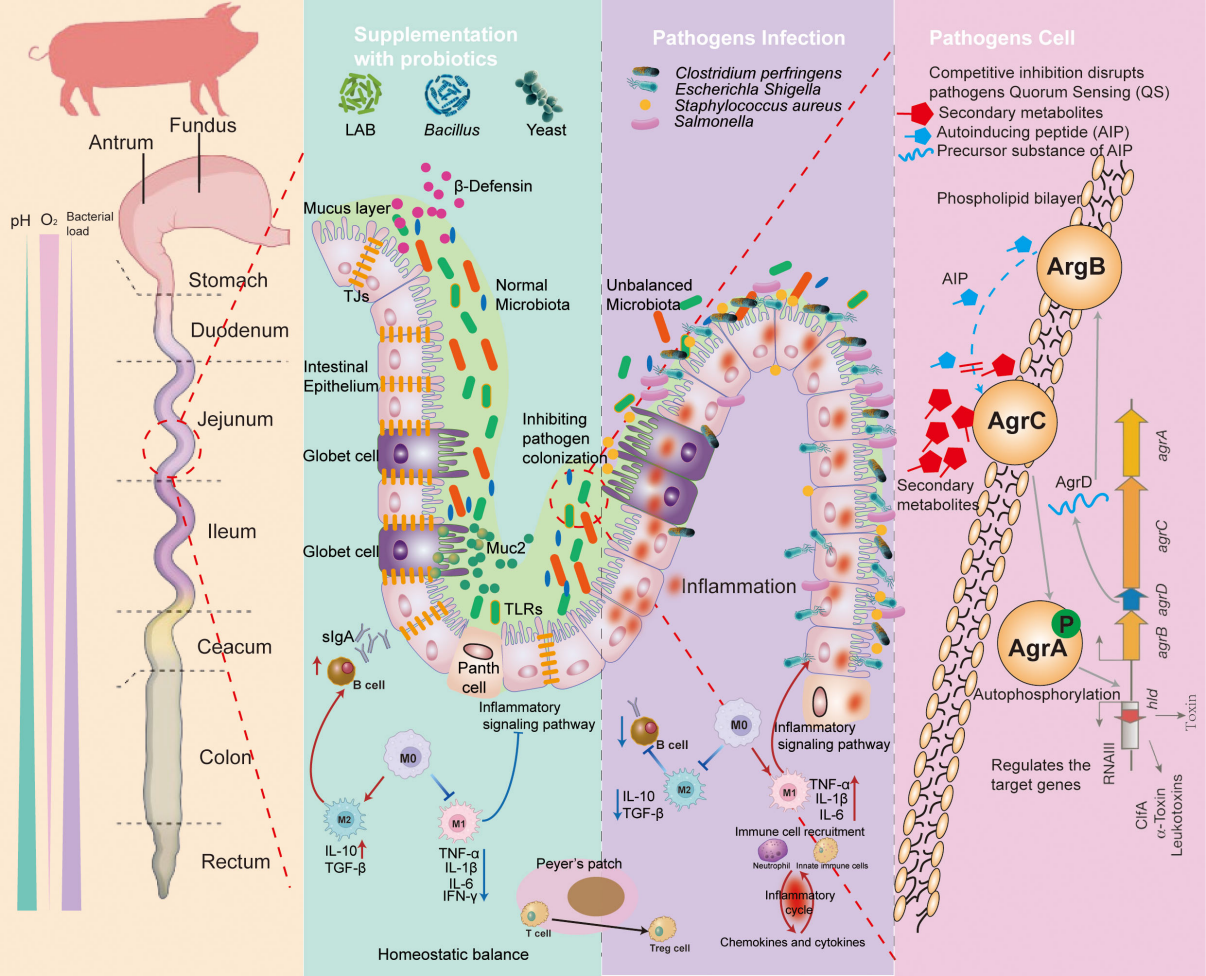
The mechanisms of probiotic prevention against the pathogen infection. Supplementation with probiotics: (1) manipulation of microbiota; (2) competitive inhibition of pathogen adhesion; (3) producing the metabolites and antimicrobial substance (such as bacteriocins, SCFAs, antimicrobial peptides, and organic acids); and (4) regulating the immune system. Pathogen infection: (1) causing microbiota disruption; (2) resulting in damage to the intestinal barrier; (3) generation of toxic substances into the host; and (4) triggering inflammatory responses. Pathogen cell: probiotics prevent the colonization of pathogens by producing secondary metabolites that block the transmission of quorum sensing (QS) systems of pathogens, leading to a reduction in the proliferation. TLRs, toll-like receptors; MUC2, mucin 2; TJs, tight junctions; DC, dendritic cell; TGF-b, transforming growth factor-b; TNF, tumor necrosis factor; IL, interleukin; sIgA, secretory immunoglobulin A; AMPs, antimicrobial peptides; AIP, autoinducing peptide.

The mechanism of probiotics in preventing pathogens is summarized in **Figure 2**. Firstly, probiotics are able to compete directly with pathogenic bacteria for ecological niches and reduce by pathogen colonization. Secondly, appropriate probiotics can be the immune activators to prevent pathogen-related inflammation via multiple signaling pathways. Thirdly, the surface molecules and metabolites of probiotics may modulate piglets’ gut barrier function or directly reduce the load of pathogenic bacteria, for example, by inhibiting the QS system, disrupting the bacterial cell membrane structure, or lowering intestinal pH. 

## Conclusion and future perspectives

This review has provided an extensive examination of the role of probiotics in maintaining and enhancing piglet gut health and discussed on their potential for defending against pathogenic bacterial infections and their mechanisms of action. We reviewed the studies that underscore the multifaceted benefits of different probiotics, including the modulation of gut microbiota, enhancement of gut barrier functions, stimulation of the immune system, and the production of antimicrobial compounds. Different from antibiotics, the molecular mechanisms of probiotics are complex and involve direct interactions with pathogens and modulation of host immune responses. Therefore, probiotics not only have considerable ability in the application of pathogen infection prevention in piglets, but also have unique advantages for antibiotic substitution.

From the available research, probiotics for prevention, treatment, or a combination may be the way forward. There are several areas of research and development that promise to advance the application of probiotics in piglet gut health. Strain Selection and Development: continued research is needed to identify and develop novel probiotic strains with enhanced efficacy against a broad spectrum of pathogens. *In Vitro* and *In Vivo* Experiments: screened probiotics that can stably colonize and efficiently display various functions (such as secreting antibacterial substances, improving the host immune system, and competing for ecological niches with pathogens) need to be verified. Personalized Probiotic Therapies: exploring the potential for personalized probiotic therapies tailored to the specific needs of individual piglets based on their unique gut microbiota profiles. Synergistic Approaches: investigating the synergistic effects of probiotics when combined with other interventions such as prebiotics, postbiotics, and phytobiotics to enhance gut health. Additionally, using the metabolites produced by probiotics directly may be a viable and efficient strategy to prevent or treat pathogen infection in piglets.
